# Monitoring of type 2 diabetic patients during the ‘Covid 19’ pandemic by teleconsultation

**DOI:** 10.1016/j.amsu.2022.103689

**Published:** 2022-04-29

**Authors:** O. Elmehraoui, S. Berrabeh, N. Messaoudi, N. Derkaoui, D. Zerrouki, S. Rouf, H. Latrech

**Affiliations:** a Department of Endocrinology-Diabetology and Nutrition, Mohammed VI University Hospital, Oujda, Morocco; b Faculty of Medicine and Pharmacy, University of Mohammed First, Oujda, Morocco; c Department of Epidemiology, Clinical Research and Public Health, University of Mohammed First, Oujda, Morocco

**Keywords:** Teleconsultation, type 2 diabetes, Covid-19 pandemic, Glycemic control, WHO, World health organization

## Abstract

**Introduction:**

Glycemic control in type 2 diabetic patients is a constant challenge. The objective of our study is to evaluate glycemic control in type 2 diabetic patients since the start of the "Covid-19" pandemic by comparing their glycemic and degenerative profiles in the pre-confinement,per and post-confinement periods.

**Patients and methods:**

This is a descriptive and analytical,cross-sectional study,bringing together 720 type 2 diabetic patients followed by teleconsultation at the endocrinology,diabetology and nutrition department,from mid-March to mid-October 2020.

**Results:**

The mean age of the patients was 62.5 ± 11.3 years, with a sex ratio (M / F) of 0.71,the average duration of diabetes was 10.6 ± 7, 4 years.The mean pre-lockdown Hba1c level was 8.5±1.9%.Only 137 patients have been able to do the HBa1c test since the start of lockdown,which averaged 8.4 ± 1.8%.Regarding the degenerative complications installed after containment,4.4% of the patients presented an acute coronary syndrome,2.2% a cerebrovascular accident,1.4% developed a stage 2 chronic renal disease,and a foot injury appeared in 5.1% of patients while no patient developed diabetic retinopathy among those who performed ophthalmic evaluation.In our series,the death rate was estimated at 1.8%.Drug therapy was adjusted in the majority of patients with initiation of insulin therapy in 7.2% of patients and intensification of insulin doses in 9.4% of patients.

**Conclusion:**

At the heart of the pandemic Covid-19 crisis,teleconsultation has taken an essential place in the strategy of access to care, particularly the monitoring of chronic diseases,the results of our study are similar to those of other studies published during this pandemic.

## Highlights

• Glycemic control in type 2 diabetic patients is a constant challenge, particularly in a pandemic situation.
• At the heart of the Covid-19 pandemic crisis, teleconsultation has taken on an essential place in the access to care strategy.
• In our study, the mean Hba1c level did not increase during the teleconsultation period.

## 1 Introduction

Since the discovery in December 2019, in Wuhan, China, of a group of people with pneumonia of unknown cause and the identification of the new coronavirus "SARS-CoV2" as responsible for this pneumonia currently called ‘Covid-19’, the whole world is facing this new progressive, serious and highly contagious with community transmission infection [1,2]. It was declared a pandemic by the World Health Organization (WHO) on March 11, 2020 [3]. As of October 15th^,^ 2020, 38,966,868 cases have been reported worldwide and 1,098,937 people have died of it [4], with 163,650 confirmed cases in Morocco including 6,087 in the eastern region [5].

Some studies showed that SARS-CoV-2 or Covid-19 infection seems more severe in the elderly and with co-morbidities, in particular diabetes mellitus, hypertension, cardiovascular pathologies, obesity, kidney disease, and chronic respiratory disease [6]. Thus, diabetes mellitus is considered to be a risk factor for the progression and poor prognosis of Covid-19 [1,7].

In the light of these findings, and given the state of health emergency in Morocco, a strategy for reorganizing hospitals has been developed, limiting scheduled hospitalizations and reserving most of the bed service capacity for emergency management and of cases with Covid-19. To best adapt to this situation, we have decided to adopt a teleconsultation program to ensure continuity of care for the benefit of our diabetic patients. Despite the difficulties of managing the teleconsultation and the burden of the task, the medical team provided it with courage and dedication.

The objective of our study is to assess glycaemic control in type 2 diabetic patients during the Covid- 19 pandemic by comparing their glycaemic and degenerative profiles in the pre-confinement, per and post-confinement period. The secondary objective is to appreciate the utility and the limitations of teleconsultation as an alternative means of follow-up in type 2 diabetic patients.

## 2 Patients and methods

### 2.1 Study design

**Fig. 1:**
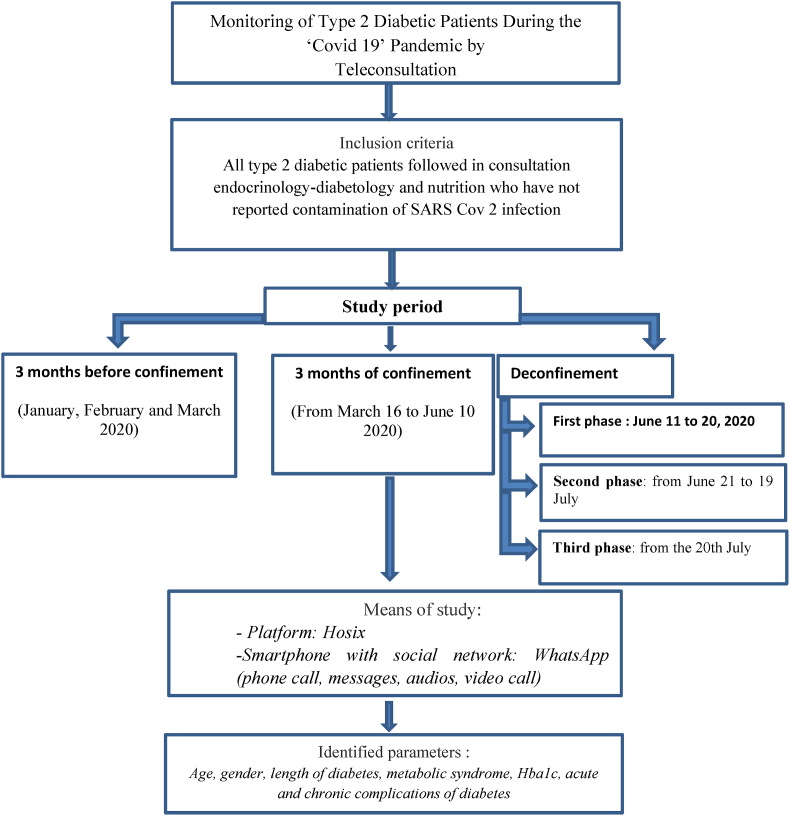
The general diagram showing the design of the study.

This is a descriptive and analytical study, with cross-sectional data collection, on insufficiently controlled and complicated type 2 diabetic patients monitored via teleconsultation provided by our team of department of endocrinology, diabetology and nutrition. Teleconsultation debuted at the time of declaration of the state of health emergency by the Moroccan authorities, since mid-March 2020 and at present given the limitation in the number of daily face-to-face consultations imposed by our hospital structure (Fig. 1).

### 2.2 Study sample

**Fig. 2:**
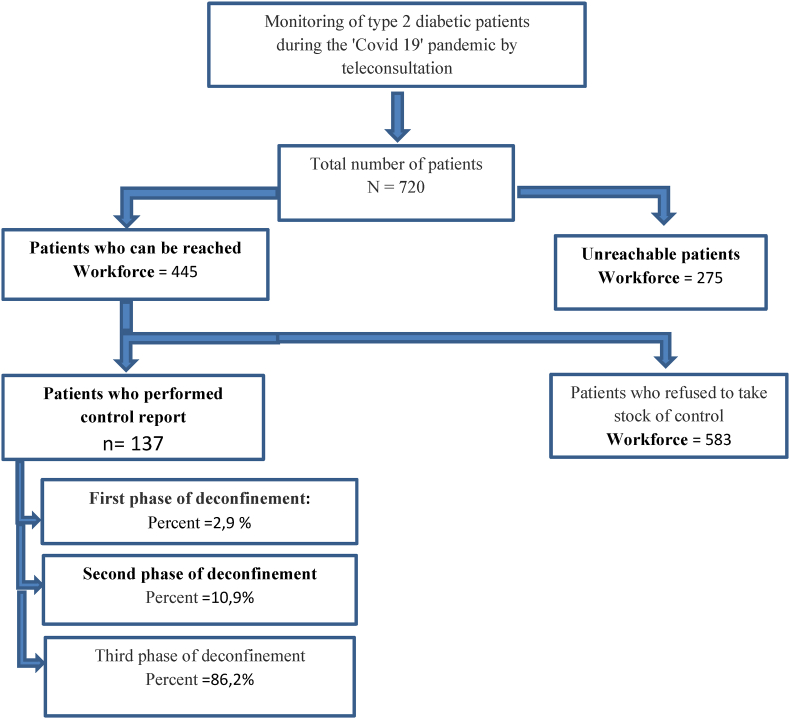
Detailed diagram showing the patients included in our study.

We contacted 720 type 2 diabetic patients during the study period, of which 61.8% were reachable, while the remainder or 38.2% were unreachable on the telephone numbers noted in their medical records. Among the people who could be reached, only 137 patients were able to do their check-ups: 2.9% during the first phase of deconfinement, 10.9% during the second phase and 86.1% during the third phase, while the majority of patients refused to do their check-ups for fear of going to medical laboratories (Fig. 2).

### 2.3 Data collection

Data collection was compiled from the clinical patient diary available on the ‘Hosix’ platform in the form of computerized medical records. The patient's contact details including first and last name, patient index, telephone number were collected from the "patient history" section. The technical means used were mainly the smartphones with regular phone calls that last around 15–30 min as their main mode of communication, which can be followed up by messages and audios.

### 2.4 Study protocol: measured variables, observation methods and techniques

During the teleconsultation, the doctor must conduct a careful interview with the diabetic patient by looking for glycaemic fluctuations (the number of minor and major hypoglycaemia) by analysing the self-monitoring of blood glucose sent by the patient via the network social 'WhatsApp', thus, screening for signs pointing to complications such as angina, claudication, subjective sensory disorder and in case of foot lesions. Video or photography could be used for inspection of the wound even if teleconsultation can in no way replace the clinical examination. The detection of dietary and therapeutic errors is essential in this telephone interview and when it is deemed insufficient, the patient is called for a face-to-face consultation by issuing the necessary certificate to be authorized to travel to the hospital during the period of confinement.

After the declaration of the first phase of lockdown, a metabolic and degenerative assessment was requested for these patients including the Hba1c level, the lipid profile, the renal function and the dosage of microalbuminuria as well as the ophthalmic examination. Any biological or radiological assessment carried out is sent by the patient, analysed and saved by the doctor in the medical file in order to maintain the confidentiality of patient data.

After having received all the necessary elements, a possible therapeutic adjustment is recommended, namely, a change of the molecules or their dosages, the initiation or the intensification of the insulin regimens, delivered via the same social network in the form of prescriptions. which must bear the name and first name of the patient, the date of the prescription, the name of the drug and its dosage, the duration of treatment and the signature of the prescriber.

While knowing that the education of the diabetic patient is the cornerstone of the care, part of the teleconsultation was devoted to dietetic and therapeutic advice which are individualized for each patient, relating to a healthy and balanced diet, a regular physical activity at home (dancing, aerobics, cycling, treadmill, etc.), muscle strengthening and stretching exercises, not to mention a reminder of preventive measures against Covid-19 and with emphasis on influenza and pneumococcal vaccination.

### 2.5 Statistical analysis

Statistical analysis was performed by SPSS version 21 software. Descriptive statistics were mentioned for all variables: means, percentages and standard deviation. The McNemar test was used to compare the mean values. For all statistical tests, a p-value less than 0.05 was considered statistically significant. Our study has been reported following the STROCSS 2021 criteria [8].

## 3 Results

### 3.1 Characteristics of the population

The mean age of the patients was 62.5 ± 11.3 years, with a sex ratio (M/F) of 0.71, the average duration of diabetes was 10.6 ± 7.4 years. Arterial hypertension was noted in 20% of patients, dyslipidaemia and metabolic syndrome were observed in 19% and 21.2% of cases, respectively.

### 3.2 The results of the study

Before the lockdown period, 6.9% of the patients studied had already had at least one decompensation of their diabetes, 17% presented minor episodes of hypoglycaemia, to due to one hypoglycaemia per week in 5.5% of patients. Degenerative complications were found in 80% of patients, 34.4% were followed for macrovascular and 46.6% had at least one of the microvascular complications (Table 1).

**Table 1:** The macro and micro vascular complications found in patients before confinement and during teleconsultation.

macro and micro vascular complications		Before confinement	During the teleconsultation
Macrovascular complications	Ischemic heart disease	31,4%	35,8%
	cerebrovascular accident	6,2%	8,4%
	Lower extremity arterial occlusive disease	11,6%	11,6%
Microvascular complications	Diabetic retinopathy	21,2%	21,2%
	Diabetic nephropathy	22,6%	24%
	Diabetic feet	14,5%	19,6%

The average level of Hba1c before containment was 8.5 ± 1.9%. Only 22.7% of patients had reached treatment goals in HbA1c. During the confinement period, the mean Hba1c was 8.4 ± 1.8% showing no statistically significant difference (p = 0.61) when comparing the mean Hba1c levels before and after confinement.

During the teleconsultation, it was found that 13.1% of the patients installed the complications of diabetes, 4.4% presented an acute coronary syndrome, 2.2% a cerebrovascular accident and concerning a Lower extremity arterial occlusive disease, none of the patients reported intermittent claudication. Only 1.4% of patients developed stage 2 chronic renal disease, and Feet lesion occurred in 5.1% of patients, while no patient developed diabetic retinopathy among those who developed an ophthalmic examination. In our series, the death rate was estimated at 1.8%. Drug therapy was adjusted in the majority of patients. Insulin therapy was initiated in 7.2% of patients and insulin doses were intensified in 9.4% of patients (Table 2).

**Table 2:** The treatment regimen of type 2 diabetic patients before and after confinement.

		Before confinement	After confinement
		Percentage	Percentage
Oral antidiabetics	Monotherapy	11.7%	10.2%
	Dual therapy	15.3%	8.8%
Insulin therapy	Bed time	22.6%	20.4%
	two mixed	23.4%	24.8%
	two mixed + fast	6.6%	8.8%
	Basal bolus	20.4%	26.3%

## 4 Discussion

Diabetes and hyperglycemia impair innate immunity, therefore, diabetic patients clearly seem to run an increased risk of developing severe and critical forms of Covid-19 infection [9,10], a finding confirmed by several studies [[11], [12], [13]].

Faced with this pandemic, several decisions have been taken concerning the organization of the Moroccan health system in order to facilitate access to care for patients with chronic diseases, in particular diabetes, while limiting the risk of contamination [14], hence the interest of adopting another means of communication in order to maintain regular monitoring of these vulnerable patients.

Telemedicine is the practice of medicine carried out remotely using information and communication technologies [15], it covers several fields ranging from teleconsultation to teleexpertise [16]. In Morocco, there are three laws which govern the practice of medical tele-medicine, first of all the law 131-13 relating to the practice of medicine which made it possible from 2014 to define and integrate telemedicine into the health system [17], then in July 2018, decree 2-18-378 which delimits the regulatory outlines of all tele-medical acts and finally, and given the sensitivity of patient health data, law 09-08 relating to the confidentiality of data [18].

During this pandemic, the National Council of the Order of Doctors allowed the use of teleconsultation to ensure remote monitoring of patients, especially those with chronic diseases or cancer, the elderly and pregnant women.

Teleconsultation puts patients in contact with their treating doctors in the comfort of their homes. Interviews and electronic prescription delivery have enabled thousands of patients to continue their care without exposing themselves or increasing the risk of contamination. Reporting the experience of the source of the pandemic, in January 2020 China activated a multimodal telemedicine network in Sichuan province, it synergizes a newly established 5G service, a smartphone application and a telemedicine system that is proven to be feasible, acceptable and effective in enabling significant improvement in health care outcomes [19]. The same, video consultations have been encouraged and extended to reduce the risk of transmission, particularly in the United Kingdom [20], in the United States [21], thus, other telemental health services have been inaugurated in China. [22] and in Australia [23].

Indeed, analysis of the results of our study showed that the mean Hba1c level did not increase as expected during the teleconsultation period. Our results are similar to those of other studies reporting the experience of teleconsultation as a means of monitoring diabetic patients. In fact, in a study conducted in China involving a population of type 2 diabetic patients (n: 3514) who benefited from teleconsultation, a reduction in HbA1c of 0.37% (p < 0.001) was noted [24]. Same result obtained by Flodgren et al. by objectifying a reduction in HbA1c of 0.31% (p < 0.001) in the patients [25]. In a recently published meta-analysis of 46 studies including patients with type 2 diabetes (n: 24,000). There was an overall mean reduction in HbA1c of (0.01%–1.13%) [26].

Likewise, the occurrence of complications, especially degenerative ones, was not so affected. The degenerative complications that occurred in our patients could be due to the natural progression of the diabetic disease, but the conditions of confinement also represent potential risk factors that would be associated with poor glycaemic control like sedentarity lifestyle. In fact, a large part of the population has been forced to work from home, with two consequences; a loss of muscle mass and weight gain which led to a decrease in insulin sensitivity. The challenge also lies in the increased calorie intake compared to daily energy expenditure. The boredom and the stress generated by confinement can also be a factor of glycaemic imbalance.

The results of our study are limited by several factors that must be taken into consideration; the number of patients who underwent a metabolic and degenerative assessment was limited; the short duration of the study in a very particular context of major health crisis. On the other hand, we were faced with considerable difficulties during this study, in particular the interviews and dietary and therapeutic education with illiterate patients, without forgetting the technical problems which are known for telemedicine, namely the problems of connection, video resolution and sound.

While knowing that the normal consultation of a patient with type 2 diabetes is very heavy, we must not forget that teleconsultation is even more so. These results are achieved thanks to the valuable work and commitment of the medical team of the diabetology and nutrition endocrinology service, despite the unfavourable conditions experienced during the period of confinement by the patients and which all promote glycaemic imbalance, including the sedentary lifestyle, change in eating behaviour [27], decrease in the purchasing power of families which in particular limits the performance of daily self-monitoring of blood glucose.

## 5 Conclusion

At the heart of the Covid-19 pandemic crisis, teleconsultation has taken on an essential place in the access to care strategy since it is a validated and recommended tool for monitoring diabetic patients. Our experience noted general satisfaction and the teleconsultation was well received by our patients. However, data remains limited and further studies are needed to confirm the long-term impact of teleconsultation.

## Ethical approval

This is a cohort study that does not require a formal ethical committee approval. Data were anonymously registered in our database. Access to data was approved by the head of the department.

## Sources of funding

This research was not funded.

## Consent

A written informed consent was obtained from the patients for publication of this cohort study and accompanying images. A copy of the written consent is available for review by the Editor-in-Chief of this journal on request.

## Author contributions

Dr. Elmehraoui Ouafae wrote the manuscript.

Dr. Berrabeh Soumiya helped in writing and exploitation of patient data.

Dr. Elmessaoudi Najoua helped in writing and exploitation of patient data.

Dr. Derkaoui Nada helped in exploitation of patient data.

Dr. Dounia Zerrouki helped in exploitation of patient data.

Pr. Siham Rouf helped in writing, supervised the redaction and revised the manuscript.

Pr. Hanane Latrech helped in writing, supervised the redaction, revised and approved the final draft for publication.

All authors approved the final version of the manuscript.

## Registration of research studies

Name of the registry: researchregistry7700
Unique Identifying number or registration ID: researchregistry7700
Hyperlink to your specific registration (must be publicly accessible and will be checked): https://www.researchregistry.com/browse-the-registry#home/registrationdetails/621ff5fb00f531001e7d5b6c/

## Guarantor

Professor Hanane Latrech.

## Declaration of competing interest

The authors declare no conflicts of interest.
